# Testosterone aggravates cerebral vascular injury by reducing plasma HDL levels

**DOI:** 10.1515/biol-2020-0107

**Published:** 2020-12-31

**Authors:** Tao Jin, Lu Wang, Dongbo Li, Tao Yang, Yuefei Zhou

**Affiliations:** Department of Neurosurgery, Ankang Central Hospital, Ankang 725000, People's Republic of China; Department of Neurosurgery, Xijing Hospital, Fourth Military Medical Hospital, Xi’an 710032, Shanxi, People's Republic of China

**Keywords:** intracranial aneurysms, oxidative stress, inflammation, macrophage, testosterone propionate

## Abstract

Testosterone is often used to improve the physiological function. But increased testosterone levels affect blood lipids and cause inflammation and oxidative stress, which are risk factors for vascular diseases. This study aimed at investigating the effects of testosterone on cerebral vascular injury using an established intracranial aneurysm (IA) model. Sixteen-week-old female C57Bl/6 mice were subcutaneously infused with testosterone propionate (TP; 5 mg/kg day) or plain soybean oil (controls) for 6 weeks. After 2 weeks of treatment, mice were given angiotensin II-elastase for another 4 weeks. The results showed that TP significantly increased cell apoptosis and reactive oxygen species production in cerebral artery, together with increases in plasma tumor necrosis factor-α (TNF-α) levels and in urinary 8-isoprostane levels. Plasma assays showed that 2 weeks after TP or soybean oil administration, the high-density lipoprotein (HDL) level was higher in the TP group than in controls. *In vitro* studies showed that testosterone increased TNF-α and monocyte chemotactic protein-1 mRNA and protein expression levels in RAW 264.7 macrophages. In summary, by reducing the HDL level, TP aggravates cerebral artery injury by increasing cell apoptosis, inflammation, and oxidative stress.

## 1 Introduction

Sex hormones are usually administered as therapeutics in patients with gonadal dysfunction or in transgenders. The levels of sex hormones can affect the development of vascular diseases, such as abdominal aortic aneurysms (AAAs) and intracranial aneurysms (IAs) [1,2]. However, the nature of testosterone effects on vascular diseases is controversial. For example, several studies indicated that testosterone protects against atherosclerosis and cardiometabolic syndrome [3,4,5]. On the contrary, some studies hold the opposite view that testosterone and its derivatives exert toxic effects on the cardiovascular system [6,7]. Animal experiments confirmed the mixed side effects of testosterone. Male mice castration improved angiotensin (Ang) II-induced AAAs [8]. Inhibition or genetic deletion of the androgen receptor decreased the growth of AAAs [9]. Testosterone administration increased the abundance of Ang II type 1A receptor mRNA in abdominal aorta and intensified Ang II-induced atherosclerosis and increased the prevalence of AAAs in neonatal female mice, but not in neonatal male mice [10]. The results of these studies indicated that an elevated level of testosterone may represent a risk factor of vascular injury.

Testosterone exerts several physiological effects, i.e., it decreases high-density lipoprotein (HDL) and increases low-density lipoprotein (LDL) levels [11,12,13]. According to a widespread opinion, a decrease in HDL and an increase in LDL levels contribute to vascular diseases through the activation of inflammatory cells. Clinical studies found that blood lipid content impacted on the formation and rupture of IAs [14,15]. High doses of testosterone could also cause inflammation and oxidative stress [16,17]. Both factors entail a high risk of IA development. It has been proved that many inflammatory cytokines such as interleukin (IL)-1β, tumor necrosis factor-α (TNF-α), and IL-6 associate with IA rupture [18]. Moreover, it is widely recognized that oxidative stress helps promote cerebrovascular diseases by intensifying inflammation, worsening endothelial dysfunction, and inducing cell apoptosis, thereby causing vessel wall remodeling and degradation and ultimately IA rupture [19,20]. However, more relevant research is needed to clarify the mechanisms by which testosterone might trigger the IA development [2].

This study aimed at investigating the effects of testosterone on the Ang II-elastase-induced cerebral vascular injury. We hypothesized that testosterone could advance vascular injury and inflammation by affecting blood lipid levels. The *in vivo* part of our study assessed the brain vascular injury, inflammatory factors, oxidative stress, and plasma lipids. To better understand the underlying mechanisms, *in vitro* cultured RAW 264.7 macrophages were treated with testosterone, and their TNF-α and monocyte chemotactic protein-1 (MCP-1) expression levels were measured.

## 2 Materials and methods

### 2.1 Animals

Mice were housed in a laminar flow cabinet with a half-day light and half-day dark cycle and fed on standard diet (1025; HFK, Beijing, China) and water. Mice were anesthetized using isoflurane (970-00026-00; RWD, Shenzhen, China).


**Ethical approval:** The research related to animal use has been complied with all the relevant national regulations and institutional policies for the care and use of animals and has been approved by the Animal Care and Use Committee of Ankang Central Hospital.

### 2.2 Animal model

To investigate testosterone effects on vascular injury, we used the previously described [21] Ang II-elastase mouse IA model. Sixteen-week-old female mice were divided into two groups. The treated group mice were subcutaneously infused for 6 weeks with testosterone propionate (TP; 5 mg/kg day; T101368; Aladin, Shanghai, China) dissolved in soybean oil. The control group mice were infused with plain soybean oil only. After 2 weeks of TP or plain soybean oil treatment, 18-week-old mice were administered Ang II (1,000 ng/kg/min) through an implanted osmotic mini-pump (1004, Alzet pump; Durect, Cupertino, USA) and injected with 17 mU elastase (E8210; Solarbio, Beijing, China) into the basal cistern on the right side.

### 2.3 Histology

At termination, mice were perfused with 4% paraformaldehyde (PFA). Next, sampled vascular tissues were fixed in 4% PFA for 24 h and then embedded in optimal cutting temperature (OCT) compound (4583; Sakura, Torrance, USA). OCT samples were sliced to 8 µm (CM1950; Leica, Wetzlar, Germany). Slides were stained using a terminal deoxynucleotidyl transferase dUTP nick-end labeling (TUNEL) kit (FA201; Transgen Biotech, Shanghai, China) for the cell apoptosis assay. To assess reactive oxygen species (ROS), we used the dihydroethidium (DHE) kit (BB47051; Bestbio, Shanghai, China). Pictures were taken under a microscope system (Axiocam 503; Zeiss, Jena, Germany; X-Cite 120Q, Excelitas, Waltham, USA).

### 2.4 Plasma TNF-α

At termination, blood was drawn from the left ventricle. Plasma was isolated by spinning at 3,000 rpm for 10 min. TNF-α was measured via an enzyme-linked immunosorbent assay (ELISA) kit (SEKM-0034-96T; Solarbio, Beijing, China) according to the manufacturer’s instructions.

### 2.5 Urine test

Before termination, a 24-h urine collection from metabolic cages was performed. To measure oxidative stress, we assayed 8-isoprostane (ELISA JL40844; Jianglai, Shanghai, China), a biomarker of oxidative stress. Creatinine was assayed as the reference compound (ELISA JL20489; Jianglai, Shanghai, China). The assays were performed following the instructions provided by the manufacturer. OD values were measured at 450 nm in a microplate reader (Epoch, BioTek, Winooski, USA).

### 2.6 Plasma lipids measurement

After 2 weeks of TP or plain soybean oil treatment, mice were sacrificed, and blood samples were drawn from their left ventricles. Plasma samples were collected after centrifugation and stored at −80°C. Plasma HDL, LDL, and triglycerides were measured by HDL (ml037767), LDL (ml063218), and triglycerides (ml063270) ELISA kits, respectively, that were bought from MLBio (Shanghai, China).

### 2.7 Cell culture

The RAW 264.7 murine macrophage cell line (SCC-211800; Solarbio, Beijing, China) was grown in high glucose Dulbecco’s Modified Eagle Medium (Hyclone, Logan, USA) supplemented with 10% FBS (13011-8611; Sijiqing, Hangzhou, China) and 1% penicillin–streptomycin. Cells were kept at 37°C in air with 5% v/v CO_2_ and subcultured every 48–72 h. Fifty thousand cells were seeded into each well of 6-well plates for 12 h and then incubated in serum-free medium for 24 h. After the addition of testosterone (10^−7^ mol/L; Aladdin, Shanghai, China), the cells were cultured for another 24 h to assess the hormone effects on macrophages. To remove cell debris, cell-conditioned media were centrifuged at 2,500 × *g* for 5 min and the supernatants were stored for further use. TNF-α and MCP-1 protein expression levels were measured by ELISA (SEKM-0108-96; Solarbio, Beijing, China). Cultured cells were collected to assess mRNA expression.

### 2.8 qRT-PCR

Total RNA was isolated using TRIzol (9109; Takara, Kusatsu, Japan). The cDNA was prepared by PrimeScript RT reagent Kit (RR047A; Takara, Kusatsu, Japan). qPCR was performed on an ABI-7300 apparatus (ABI, Foster, USA) using SYBR Green (B21203; Bimake, Shanghai, China). All steps were performed according to the manufacturer’s instructions. Primers: *Tnf-*α, forward, 5′-ACGTCGTAGCAAACCACCAA-3′, reverse, 5′-GCAGCCTTGTCCCTTGAAGA-3′; *Mcp-1*, forward, 5′-CCAGCCTACTCATTGGGATCA-3′, reverse, 5′-CTTCTGGGCCTGCTGTTCA-3′; *Gapdh*, forward, 5′-AGGTCGGTGTGAACGGATTTG-3′, reverse, 5′-GGGGTCGTTGATGGCAACA-3′. *Gapdh* was the reference gene used.

### 2.9 Statistical analysis

Results were expressed as mean ± SD. Group mean differences were evaluated by Student’s *t*-test using GraphPad Prism 6 (San Diego, USA). A *P* value of <0.05 was considered statistically significant.

## 3 Results

### 3.1 TP aggravated Ang II-induced apoptosis and ROS production in a mouse IA model

**Figure 1:**
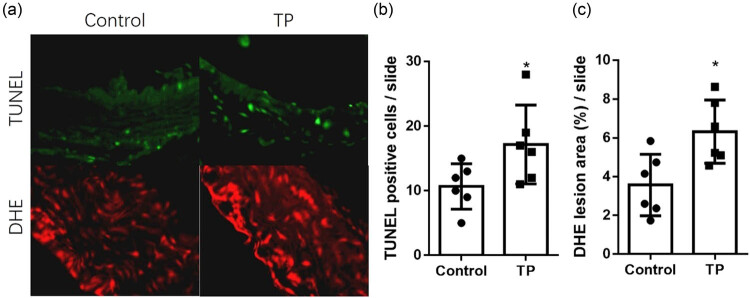
TP aggravated vascular lesions in a mouse IA model. (a) Representative pictures of a cerebrovascular wall stained with DHE and TUNEL separately. (b) TUNEL staining showed that TP increased cell apoptosis in the blood vessel (positive cells/slide; control, 10.67 ± 3.502 vs TP, 17.17 ± 6.113; *n* = 6). (c) DHE staining showed that TP increased ROS production in the blood vessel wall (lesion area %/slide; control, 3.573 ± 1.589 vs TP, 6.323 ± 1.631; *n* = 6). *P < 0.05. TP, testosterone propionate.

To investigate the effects of testosterone on vascular cell apoptosis, the TUNEL assay was applied to cerebral blood vessel slides. Results showed that TP significantly increased cell apoptosis in cerebral blood vessels (Figure 1a and b). DHE staining showed that TP significantly increased ROS production in cerebral blood vessel sections (Figure 1a and c). It means that TP aggravated Ang II-induced cerebral vascular injury.

### 3.2 TP increased the plasma inflammatory marker TNF-α and the urinary oxidative stress marker 8-isoprostane

**Figure 2:**
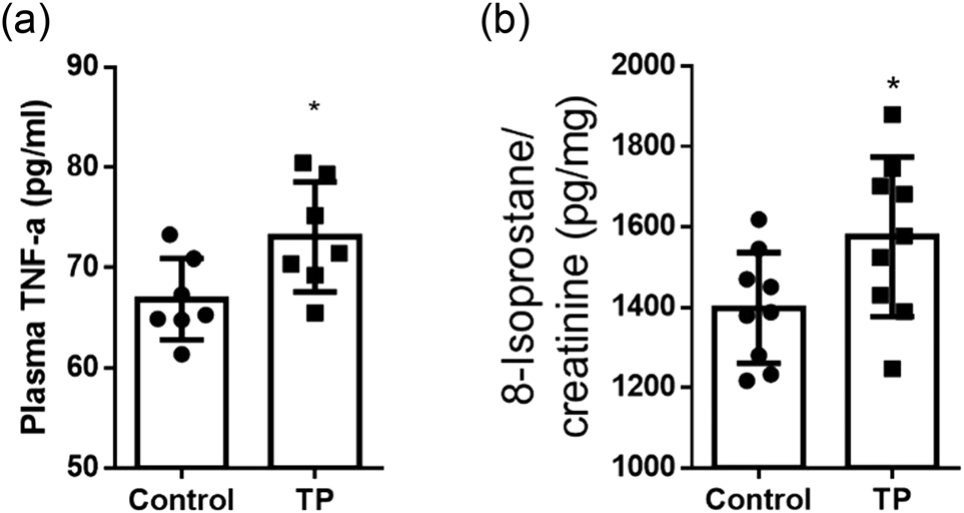
TP increased inflammation and oxidative stress. (a) Plasma TNF-α level (pg/mL; control, 66.85 ± 4.050 vs TP, 73.06 ± 5.468; *n* = 7). (b) Urinary 8-isoprostane/creatinine ratio (pg/mg; control, 1398 ± 137.7 vs TP, 1575 ± 198.2; *n* = 9). *P < 0.05. TP, testosterone propionate.

Ang II promotes inflammation and oxidative stress [22]. To confirm the effects of TP on inflammation and oxidative stress, the plasma TNF-α and urinary 8-isoprostane levels were measured. Compared to the control group, TP significantly increased the plasma TNF-α level (Figure 2a) and the urine 8-isoprostane/creatinine ratio (Figure 2b).

### 3.3 TP decreased HDL plasma levels in mice

**Figure 3:**
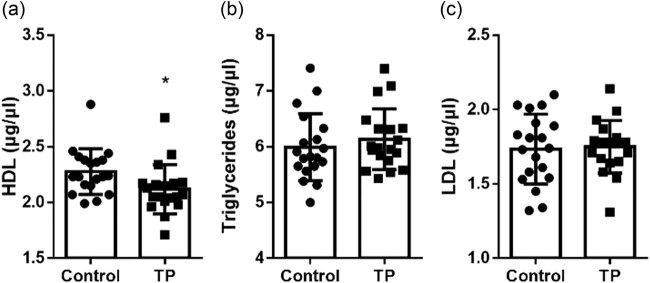
Plasma lipid levels after 2 weeks of TP or plain soybean oil (control) treatment. (a) TP significantly decreased plasma HDL level (μg/μL; control, 2.276 ± 0.2050 vs TP, 2.120 ± 0.2207; *n* = 19). (b and c) TP did not change the levels of plasma triglycerides (μg/μL; control, 5.991 ± 0.6016 vs TP, 6.132 ± 0.5458; *n* = 19) and LDL (μg/μL; control, 1.734 ± 0.2345 vs TP, 1.749 ± 0.1771; *n* = 19). *P < 0.05. TP, testosterone propionate.

To detect the effects of TP on the regulation of plasma lipid levels in mice, after 2 weeks of treatment with TP or plain soybean oil, the mice were sacrificed and blood samples were drawn from them. The results showed that TP significantly decreased HDL plasma levels in mice (Figure 3a) but did not change LDL and triglyceride levels (Figure 3b and c).

### 3.4 TP increased TNF-α and MCP-1 mRNA and protein expression levels in RAW 264.7 macrophages

**Figure 4:**
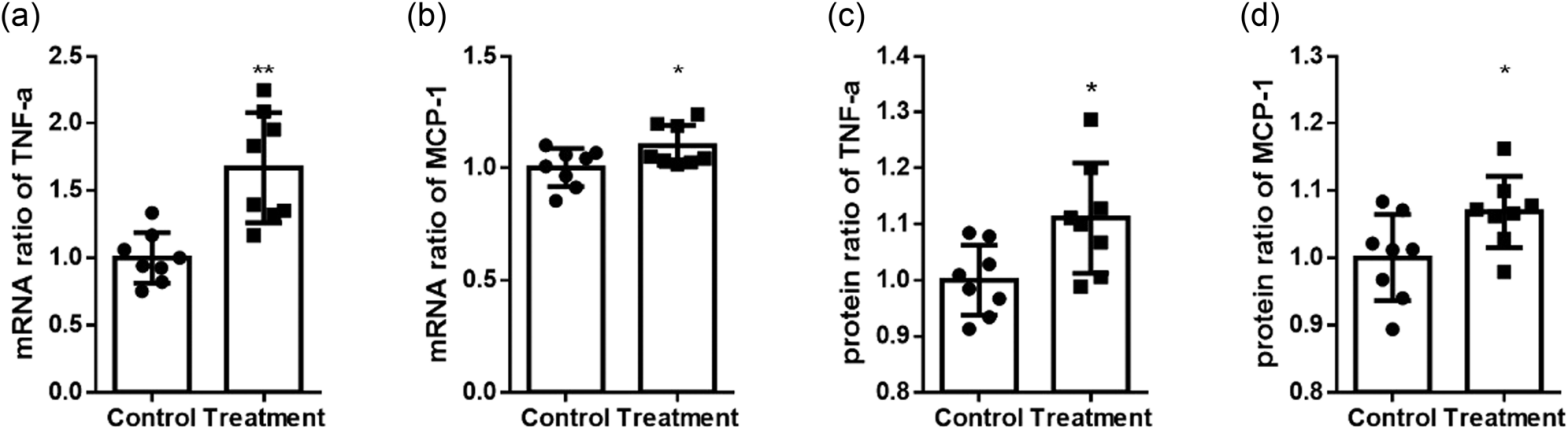
TP increased TNF-α and MCP-1 levels in RAW 264.7 macrophages. (a and b) The mRNA expression levels of TNF-α (ratio; control, 1.000 ± 0.1868 vs treatment, 1.666 ± 0.4675; *n* = 8) and MCP-1 (ratio; control, 1.000 ± 0.08452 vs treatment, 1.097 ± 0.09157; *n* = 8). (c and d) The protein levels of TNF-α (ratio; control, 1.000 ± 0.06251 vs treatment, 1.111 ± 0.09776; *n* = 8) and MCP-1 (ratio; control, 1.000 ± 0.06382 vs treatment, 1.068 ± 0.05251; *n* = 8). *P < 0.05. **P < 0.01.

To investigate the pro-inflammatory effects of TP on macrophages, RAW 264.7 cells were treated with TP. qRT-PCR and ELISA results showed that TP significantly increased TNF-α and MCP-1 mRNA and protein expression levels, respectively (Figure 4a–d).

## 4 Discussion

Our present results show for the first time in an IA mouse experimental model that TP reduced plasma HDL level and increased cell apoptosis, oxidative stress, and inflammation, resulting in cerebral vascular injury.

Testosterone-induced vascular injury may be divided into several stages. At the early stage of the experiment, after 2 weeks of TP or plain soybean oil treatment, we isolated the blood before the Ang II-elastase infusion and analyzed the plasma lipids. It showed that TP decreased the HDL levels. In general, HDL is a key factor regulating vascular inflammation and macrophage infiltration. High levels of HDL can reduce macrophage infiltration and even remove infiltrated macrophages or push them toward the anti-inflammatory (M2) subtype [23]. Macrophages are the main source of oxidative stress products. Altered levels of both lipids and ROS are important causes of vascular injury. Later IA experiments in this study also confirmed this result. TP increased plasma TNF-α and urinary 8-isoprostane levels. To clarify whether testosterone directly affects the role of macrophages in vascular injury, we performed *in vitro* experiments to verify it. Consistent with our hypothesis, TP directly promoted an increased expression of the inflammatory factors TNF-α and MCP-1 in RAW 264.7 cells. These inflammatory factors can recruit additional macrophages, which may be the cause of increasing vascular inflammation intensity. Besides, macrophages can also degrade the vascular extracellular matrix (VECM) by secreting proteases, thus promoting vascular wall remodeling. Under the joint action of these factors, TP advanced the development of cerebral vascular injury.

Several previous studies have shown results that are consistent with ours but in different tissues. In AAA studies, testosterone treatment promoted aneurysm growth [24]. Inhibition or genetic deletion of the androgen receptor attenuated the AAA development, which was related to the decreased pro-inflammatory factors IL-1α, IL-6, and IL-17 [9]. These results may be explained by the fact that testosterone increased collagen deposition but decreased elastin deposition [1,25,26], leading to vascular wall remodeling. Altogether, the aforementioned results and those of this study show that high testosterone levels increase the probability of vascular injury.

In TP-aggravated cerebral vascular injury, the decrease in HDL acts as a key regulator. A clinical study showed that testosterone level is the major reason for the male–female differences in serum HDL-cholesterol levels, whereas the serum levels of total cholesterol, LDL, triglycerides, apolipoprotein B, and apolipoprotein A2 were not affected by testosterone [13]. A cohort study found that a lower HDL level in females was the only risk factor of a big size IA [14]. Another clinical study also remarkably indicated that lipid-lowering drugs and a higher HDL level are inversely associated with IA rupture, whereas LDL and total cholesterol levels were not significant under the same respect [15]. These studies further increased our knowledge of the HDL protective mechanisms against vascular injury. Therefore, we believe that the testosterone administration–aggravated brain vascular injury in our female IA model is linked to the concurrently reduced HDL levels.

Some studies held the opposite view. They suggested that testosterone exerts neuroprotective and anti-inflammatory effects in the central nervous system [2,27,28]. Also, testosterone inhibited vasospasms in subarachnoid hemorrhage model rabbits [29]. These findings may be related to the dosage and users. Most clinical studies of sex hormones are concerned with people with endocrine disorders or testosterone deficiency, especially middle-aged and older men [30,31]. Administered testosterone restores sex hormones to normal levels. There is no doubt that normal hormone levels are good for the body. But any overdose may entail potential risks such as increases in inflammation, oxidative stress, and cardiovascular disease. A study of renal ischemia–reperfusion (I/R)–induced acute kidney injury (AKI) in male rats showed that low-dose (20 µg/kg) testosterone protected against I/R AKI by reducing renal T-cell infiltration and shifting the balance of pro-inflammatory/anti-inflammatory cytokine production [32]. Higher dose of testosterone (100 µg/kg) not only did not mitigate renal injury but also increased both IL-10 and TNF-α levels. A recent review shared our point of view [7]. They pointed out that the number of testosterone prescriptions in the United States has increased in recent years. More than one-sixth of the men taking such therapy did not reach the baseline value of serum testosterone, suggesting that the patients may unnecessarily have taken testosterone. A later analysis found that testosterone may increase the cardiovascular risk. In consequence, we believe that the dose of testosterone to be administered is crucial to properly regulate physiological functions.

In summary, the administration of TP aggravates cerebral vascular injury by increasing the oxidative stress and inflammation by decreasing the HDL level in an Ang II-elastase-induced IA mice model. These results further inform us about the vascular protection mechanisms of sex hormones and their receptors, especially those related to the regulation of macrophage function and plasma HDL levels. They also supply a theoretical basis for the clinical application. It is necessary to measure the serum testosterone value before prescribing it to people who need testosterone therapy to avoid the risks of an overtreatment. Alternatively, testosterone supplements should be contraindicated in patients with a diagnosis of IA.
